# O_2 _delivery and CO_2 _production during cardiopulmonary bypass as determinants of acute kidney injury: time for a goal-directed perfusion management?

**DOI:** 10.1186/cc10349

**Published:** 2011-08-10

**Authors:** Filip de Somer, John W Mulholland, Megan R Bryan, Tommaso Aloisio, Guido J Van Nooten, Marco Ranucci

**Affiliations:** 1 Department of Interventional and Surgical Cardiology, Heart Centre, Universitair Ziekenhuis Gent, De Pintelaan 185, B-9000 Gent, Belgium; 2 Department of Clinical Perfusion Science, Essex Cardiothoracic Centre, Basildon University Hospital, Nethermayne, Basildon, Essex, SS16 5NL, UK; 3 Department of Cardiothoracic-Vascular Anesthesia and Intensive Care, IRCCS Policlinico San Donato, Via Morandi 30, I-20097 San Donato Milanese (Milan), Italy

## Abstract

**Introduction:**

Acute kidney injury (AKI) is common after cardiac operations. There are different risk factors or determinants of AKI, and some are related to cardiopulmonary bypass (CPB). In this study, we explored the association between metabolic parameters (oxygen delivery (DO_2_) and carbon dioxide production (VCO_2_)) during CPB with postoperative AKI.

**Methods:**

We conducted a retrospective analysis of prospectively collected data at two different institutions. The study population included 359 adult patients. The DO_2 _and VCO_2 _levels of each patient were monitored during CPB. Outcome variables were related to kidney function (peak postoperative serum creatinine increase and AKI stage 1 or 2). The experimental hypothesis was that nadir DO_2 _values and nadir DO_2_/VCO_2 _ratios during CPB would be independent predictors of AKI. Multivariable logistic regression models were built to detect the independent predictors of AKI and any kind of kidney function damage.

**Results:**

A nadir DO_2 _level < 262 mL/minute/m^2 ^and a nadir DO_2_/VCO_2 _ratio < 5.3 were independently associated with AKI within a model including EuroSCORE and CPB duration. Patients with nadir DO_2 _levels and nadir DO_2_/VCO_2 _ratios below the identified cutoff values during CPB had a significantly higher rate of AKI stage 2 (odds ratios 3.1 and 2.9, respectively). The negative predictive power of both variables exceeded 90%. The most accurate predictor of AKI stage 2 postoperative status was the nadir DO_2 _level.

**Conclusions:**

The nadir DO_2 _level during CPB is independently associated with postoperative AKI. The measurement of VCO_2_-related variables does not add accuracy to the AKI prediction. Since DO_2 _during CPB is a modifiable factor (through pump flow adjustments), this study generates the hypothesis that goal-directed perfusion management aimed at maintaining the DO_2 _level above the identified critical value might limit the incidence of postoperative AKI.

## Introduction

After cardiac surgery, renal function impairment is common, and acute kidney injury (AKI) has an incidence that may reach 50% according to some definitions [1]. The early mortality rate in patients with AKI is around 5% but climbs to 50% when renal replacement therapy is required [2-4]. Various factors related to cardiopulmonary bypass (CPB) have been implicated as possible determinants of AKI. They include CPB duration [2,5,6], low perfusion pressure [7], low pump flow [7,8], severe haemodilution [8-11] and low oxygen delivery (DO_2_) [8].

In 2005, in a retrospective series, we demonstrated that a lowest (nadir) DO_2 _level of 272 mL/minute/m^2 ^during CPB was independently associated with acute renal failure and peak postoperative serum creatinine levels [8]. Subsequently, we identified that DO_2 _levels < 260 mL/minute/m^2 ^during CPB were associated with increased lactate formation [12] and that hyperlactatemia was associated with decreased DO_2 _levels and an increased CO_2 _production (VCO_2_) during CPB, with critical values settled at a VCO_2 _> 60 mL/minute/m^2 ^and a DO_2_/VCO_2 _ratio < 5.0 [13].

These data generate the hypothesis that when the DO_2 _during CPB falls below a critical value (in the range of 260 to 270 mL/minute/m^2^), organ dysoxia may be triggered, with consequent tissue acidosis leading to increased VCO_2_, and that this mechanism may be a determinant of impaired postoperative renal function.

Despite this information, routine measurement of DO_2 _and VCO_2 _is still not the standard of care during CPB. The quality indicators used for CPB management are wide-ranging and are mostly 'stand alone' parameters. There is little or no evidence in the literature to suggest that goal-directed perfusion pressure using current quality indicators [14] influences clinical outcomes. The finding that low DO_2 _during CPB is an independent determinant of postoperative AKI is still based on a single-centre observation.

In the present dual-centre large series of patients where these values were routinely monitored during CPB, we investigated the hypothesis that DO_2 _and VCO_2 _during CPB might be independently associated with postoperative AKI.

## Materials and methods

In this retrospective analysis of prospectively collected data collected at two institutions (Heart Centre, Universitair Ziekenhuis Gent, Gent, Belgium, and Department of Cardiothoracic Surgery, Essex Cardiothoracic Centre, London, UK) DO_2 _and VCO_2 _measurement were introduced as part of standard monitoring during CPB in 2009. The primary end point of this study was the determination whether the nadir DO_2 _value and DO_2_/VCO_2 _ratio and the VCO_2 _peak value during CPB are independently associated with postoperative renal function impairment, which we defined as the peak postoperative serum creatinine value and the presence of AKI according to the AKI Network (AKIN) criteria [15]. Briefly, a patient was assigned to the AKI stage 1 group based on an increase in peak postoperative serum creatinine greater than or equal to 150% to 200% from the baseline value and to the AKI stage 2 group based on an increase in peak postoperative serum creatinine to more than 200% to 300% from the baseline value. Patients assigned to AKI stage 3 (peak postoperative serum creatinine value more than three times the baseline value) were identified but included in the AKI stage 2 group because of the predictable low rate of events. According to the AKIN criteria, the assignment of patients to the different AKI stages was based on creatinine changes only, and urine output was not considered. Creatinine changes were recorded within the first 48 hours after the operation. The secondary end point was exploring the association of the same DO_2_- and VCO_2_-related variables with general outcome measurements (length of ICU and postoperative hospital stays).

The study was approved by the local ethics committee and the institutional review boards of the two participating institutions (Local Ethics Committee Universitair Ziekenhuis Gent, Gent, Belgium, registration number B670201010162; Institutional Review Board Basildon University Hospital, Nethermayne, Basildon, Essex, SS165NL, UK), and the need for written informed consent from the patients was waived.

### Patients

Three hundred fifty-nine patients (Gent: 223; London: 136) who had surgery between September 2009 and June 2010 were admitted into the study. Entry criteria were patient age > 18 years undergoing CPB and expected CPB duration > 90 minutes. Exclusion criteria were the presence of chronic renal failure being treated with dialysis and patients who had undergone heart or heart and lung transplantation.

The patients were consecutively enrolled at both Institutions. Since only one dedicated software system was available for data collection at each Institution, some patients were not included in cases where two operations were being performed simultaneously on two suitable candidates for enrolment. This criterion accounted for a missing cases rate of 23%. The patients included in the present series were not included in the previous studies dealing with the same experimental hypothesis.

### Sample size

Power analysis was performed before retrospective data collection. On the basis of historical data from the two institutions, the postoperative AKI stage 2 rate was calculated as 12%. The ratio of patients with low DO_2 _(< 260 mL/minute/m^2^) was calculated as 35%. The study was powered to detect a double AKI stage 2 rate in patients with low DO_2_. Considering the unequal distribution of patients with low and high DO_2_, an α value of 0.05 and a β value of 0.20, the following sample size was calculated: (1) 195 patients with high DO_2 _(65%) comprising 18 patients with AKI (9%) and (2) 105 patients with low DO_2 _(35%) comprising 18 patients with AKI (17%). The total patient population included in the study was therefore between 300 and 400 patients to account for possible lack of data during the study period.

### Data collection and definitions

The following data were recorded for all patients.

1. Preoperative data: Patient demographics, baseline serum creatinine level (mg/dL), estimated creatinine clearance (measured using the Cockcroft-Gault equation), left ventricular ejection fraction (%), comorbidities (diabetes, chronic obstructive pulmonary disease, previous cerebrovascular accident), baseline haemoglobin (Hb) value (g/dL), logistic EuroSCORE and acute renal failure risk according to a validated risk score [6].

2. Perioperative data: Reoperation, type of operation, CPB duration, nadir body temperature (°C) during CPB, nadir haematocrit (HCT) level (%) (measured at the start of the CPB operation and every 20 minutes thereafter), nadir DO_2 _(mL/minute/m^2^), peak VCO_2 _(mL/minute/m^2^) and nadir DO_2_/VCO_2 _ratio during CPB, peak serum lactate during CPB and number of packed red cell (PRC) units transfused during CPB.

3. Postoperative data: Peak serum creatinine level (mg/dL), peak postoperative change in creatinine level (percentage change from baseline), estimated lowest creatinine clearance, AKI stage defined according to the AKIN criteria, number of PRC units transfused and days spent in the ICU and postoperatively in the hospital.

The number of PRC units transfused was considered as a potential risk factor for renal function impairment. Since transfusions may be the consequence rather than the cause of renal dysfunction, we considered the number of PRC units transfused before the onset of the renal dysfunction (perioperatively or soon after surgery).

### Cardiopulmonary bypass technique

All patients were treated with moderately hypothermic CPB (27°C to 36°C). The CPB circuit consisted of an Avant oxygenator (Sorin Group, Mirandola, Italy), an HVR Evo hard-shell reservoir (Sorin Group) in London and a closed CVR 1200 soft-shell reservoir (Sorin Group) in Gent, Belgium. The hardware consisted of a Stöckert S5 heart-lung machine (Sorin Group) and a Stöckert Heater Cooler System 3T (Sorin Group).

Priming solutions consisted of 1,400 mL of Hartmann's solution and 10,000 IU of heparin (London site) and 1,200 mL of gelatine (1,000 mL), 15% mannitol (200 mL) and 2,500 IU of heparin (Gent site). No hydroxyethyl starch was used for priming the circuit or as additional fluid during CPB. Cardioplegic solution was a 4:1 ratio of cold blood cardioplegia (St. Thomas's solution) at an induction dose of 1,000 mL, with subsequent 200-mL doses every 20 minutes of (London site) and 800- to 1000-mL single-dose Saint Thomas II crystalloid cardioplegia with additional doses if considered necessary by the surgeon (Gent site). Pump flow was settled at 2.4 to 2.8 L/minute/m^2 ^at the outset of CPB and subsequently adjusted according to the patient's core temperature (usually a 20% decrease for core temperatures between 30°C and 34°C and an additional 10% decrease for core temperatures < 30°C). A Hb value less than 6 to 7 g/dL during CPB was considered the trigger point for PRC transfusion. All patients received tranexamic acid according to the routine protocol of each institution, and no aprotinin was used.

### Oxygen- and carbon dioxide-related measurements

DO_2_- and VCO_2_-related measurements were performed using a dedicated software system provided by Dideco (Sorin Group). Data were collected at 10-minute intervals during CPB. Data required to calculate DO_2 _and VCO_2 _were arterial Hb (g/dL), pump flow (L/minute/m^2^), arterial O_2 _saturation, gas flow into the oxygenator (L/minute) and expiratory CO_2 _tension (mmHg) measured at the site of the oxygenator exhaled gas port with a Datex-Ohmeda capnograph (GE Healthcare, Little Chalfont, UK)), or with a ventilator-integrated capnograph.

All blood gas data were corrected for temperature by using standard equations. The equations used to determine DO_2 _and VCO_2 _were described in a previous article [13]. Briefly, DO_2 _was calculated using the following equation:

$$\text{D}{\text{O}}_{2}\left(\text{mL}∕\text{minute}∕{\text{m}}^{2}\right)=10\times \text{pump flow (L}∕\text{minute}∕{\text{m}}^{2}\text{)}\times \text{arterial }{\text{O}}_{2}\text{content (mL}∕100\text{ mL)},$$

where arterial O_2 _content was calculated as follows:

Arterial O_2 _content (mL/100 mL) = Hb (mg/dL) × 1.34 × Hb saturation (%) + 0.003 × O_2 _tension (mmHg).

VCO_2 _was calculated using the following equation:

$$\begin{array}{l} {\text{VCO}}_{2}(\text{mL}/\text{minute}/{\text{m}}^{2})={\text{eCO}}_{2}(\text{mmHg})\times \text{Ve}\;\text{(L}/\text{minute)}\times 1,000\\ \;\;\;\;\;\;\;\;\;760\times \text{body surface area}\;{\text{(m}}^{2}\text{)}. \end{array}$$

Gas volume and flow are expressed as dry standard temperature and pressure. Adequate corrections according to body temperature and pressure saturated conditions were applied.

The nadir DO_2 _level was defined as the lowest DO_2 _value registered for at least two consecutive measurements, the peak VCO_2 _value was defined as the highest VCO_2 _level registered for at least two consecutive measurements and the nadir DO_2_/VCO_2 _ratio was defined as the lowest value registered for at least the two consecutive measurements. The normal DO_2 _and VCO_2 _values in healthy, awake subjects are usually around 500 mL/minute/m^2 ^and 100 mL/minute/m^2^, respectively. Under moderate hypothermia, anaesthesia and haemodilution during CPB, these values are always reduced to unspecified values.

### Statistical analysis

Data are expressed as means ± standard deviation (normally distributed continuous variables) or as frequencies and percentages (categorical variables). Nadir DO_2_, nadir DO_2_/VCO_2 _ratio and peak VCO_2 _during CPB were tested for association with kidney function variables using bivariate linear regression analysis (for peak postoperative serum creatinine) or bivariate logistic regression analysis (for AKI stage 1 or 2 and any kind of AKI). Other possible confounders were tested for associations with kidney function variables using the same technique. The linearity assumption was checked by exploring the decile-based distribution of the AKI stage 2 rate with spline curves. For the independent predictors of AKI, adequate cutoff values were identified using a receiver operating characteristic (ROC) curve, and the area under the curve (AUC) was used to determine the accuracy of the model. The cutoff values were chosen according to Youden's index (best sensitivity value + (specificity - 1)). For each cutoff value, the sensitivity, specificity and negative and positive predictive values were measured.

All the factors associated (*P *< 0.1) with AKI stage 2 or any kind of AKI were entered into multivariable stepwise forward logistic regression models for definition of the independent predictors of kidney function impairment to generate odds ratios with 95% confidence intervals. To avoid overfitting of the models, only one independent variable per 10 events was entered into the model. To avoid multicollinearity, variables affected by mathematical coupling were separately entered into different models. In cases of intercorrelation, the best single independent variable was chosen. For all the statistical tests, a *P *value < 0.05 was considered significant. Data analysis was performed using SPSS 13.0 statistical software (SPSS Inc., Chicago, IL, USA).

## Results

The demographics, preoperative, operative and postoperative details of the patient population are shown in Table 1. In our patient population (*N *= 359), we identified 31 patients (8.6%) with AKI stage 1 and 44 patients (12.2%) with AKI stage 2, for a total of 75 patients (21%) with any kind of AKI. Within the AKI stage 2 group, five patients (1.4%) with AKI stage 3 were included.

**Table 1:** Demographics and perioperative details of the patient population^**a**^

Factors	Patient data (*N *= 359)
Age (years) - mean ± SD	66.1 ± 13.4
Gender male - number (%)	255 (71)
Weight (kg) - mean ± SD	77.2 ± 17.5
Body surface area (m^2^) - mean ± SD	1.91 ± 0.22
Baseline serum creatinine (mg/dL) - mean ± SD	1.14 ± 0.63
Estimated creatinine clearance (mL/minute) - mean ± SD	75.6 ± 31.7
Logistic EuroSCORE - mean ± SD	9.8 ± 14.1
Acute renal failure risk score [6] - mean ± SD	1.48 ± 0.98
Diabetes - number (%)	78 (22)
Chronic obstructive pulmonary disease - number (%)	37 (10)
Previous cerebrovascular accident - number (%)	32 (8.1)
Baseline haemoglobin value (g/dL) - mean ± SD	38 ± 4.7
Reoperation - number (%)	29 (8.1)
Isolated coronary operation - number (%)	170 (47)
Coronary plus valve operation - number (%)	74 (21)
Valve operation - number (%)	80 (22)
Other operations - number (%)	35 (10)
CPB duration (minutes) - mean ± SD	108 ± 44.6
Nadir temperature (°C) during CPB - mean ± SD	30.9 ± 3.1
Nadir haemoglobin value (mg/dL) during CPB - mean ± SD	8.36 ± 1.28
Nadir hematocrit (%) during CPB - mean ± SD	25.1 ± 3.8
Nadir DO_2 _(mL/minute/m^2^) during CPB - mean ± SD	278 ± 45
Peak VCO_2 _(mL/minute/m^2^) during CPB - mean ± SD	52.5 ± 9.1
Nadir DO_2_/VCO_2 _ratio during CPB - mean ± SD	5.69 ± 1.1
Peak serum lactate (mmol/L) during CPB - mean ± SD	2.7 ± 0.98
Peak postoperative serum creatinine (mg/dL) - mean ± SD	1.56 ± 0.99
Peak postoperative creatinine change (%)- mean ± SD	39 ± 62
Nadir postoperative creatinine clearance (mL/minute) - mean ± SD	62.1 ± 32.8
Acute kidney injury stage 1 - number (%)	31 (8.6)
Acute kidney injury stage 2 - number (%)	44 (12.2)
Any acute kidney injury - number (%)	75 (21)
Packed red blood cells transfusion (U) - median (interquartile range)	1 (2)
ICU length of stay (days) - median (interquartile range)	1 (0)
Postoperative hospital stay (days) - median (interquartile range)	5 (3)

^a^CPB: cardiopulmonary bypass; DO_2_: oxygen delivery; SD: standard deviation; VCO_2_: carbon dioxide production.

The variables listed in Table 1 were tested for univariate association with the renal function outcomes (Table 2). The effect of the cardiac surgery centre on renal outcomes was analysed. Centre, patient age, diabetes, reoperation, EuroSCORE, combined coronary and valve operation, CPB duration, number of PRC units transfused and the three study variables nadir DO_2_, peak VCO_2 _and nadir DO_2_/VCO_2 _ratio during CPB were all associated with renal outcomes and were therefore admitted into the following multivariable analyses. The other perioperative variables were not significantly associated with renal outcomes.

**Table 2:** Bivariate association between perioperative factors, study variables and renal outcomes^**a**^

	AKI stage 1		AKI stage 2		Any AKI	
Variable	β coefficient	*P *value	β coefficient	*P *value	β coefficient	*P *value
Centre (1 or 2)	0.986	0.036^b^	2.034	0.001^b^	1.678	0.001^b^
Age (years)	0.049	0.011^b^	0.020	0.142	0.035	0.004^b^
Diabetes	0.762	0.057	0.346	0.345	0.611	0.037^b^
EuroSCORE	0.012	0.309	0.036	0.001^b^	0.034	0.001^b^
Reoperation	-18.9	0.998	1.313	0.003^b^	0.565	0.183
CABG and valve operation	0.709	0.085	1.103	0.001^b^	1.104	0.001^b^
CPB duration (minutes)	0.006	0.910	0.009	0.001^b^	0.008	0.005^b^
PRCs transfusion (U)	0.103	0.288	0.221	0.004^b^	0.206	0.002^b^
Lowest HCT during CPB	-0.075	0.147	-0.114	0.012^b^	-0.111	0.003^b^
Nadir DO_2 _(mL/minute/m^2^)	-0.005	0.198	-0.012	0.001^b^	-0.011	0.001^b^
Peak VCO_2 _(mL/minute/m^2^)	-0.052	0.014^b^	0.030	0.090	-0.005	0.743
Nadir DO_2_/VCO_2 _ratio	0.136	0.433	-0.408	0.010^b^	-0.192	0.119

^a^AKI: acute kidney injury; β: regression coefficient; CABG: coronary artery bypass graft; CPB: cardiopulmonary bypass; HCT: hematocrit; PRCs: packed red blood cells; DO_2_: oxygen delivery; VCO_2_: carbon dioxide production; ^b^statistically significant *P *values.

**Figure 1:**
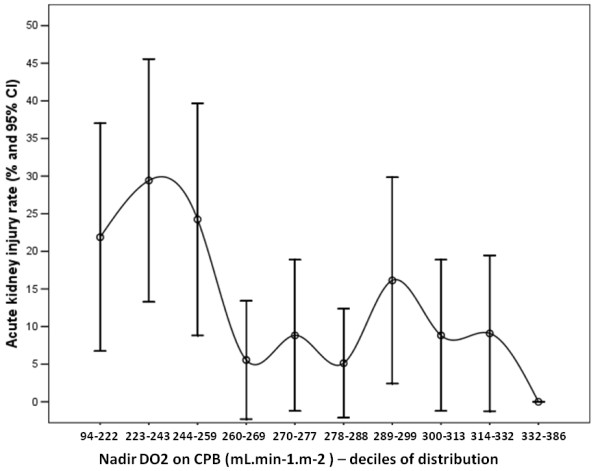
**Graph showing acute kidney injury rate according to decile distribution of nadir oxygen delivery (DO_2_) level during cardiopulmonary bypass (CPB)**.

**Figure 2:**
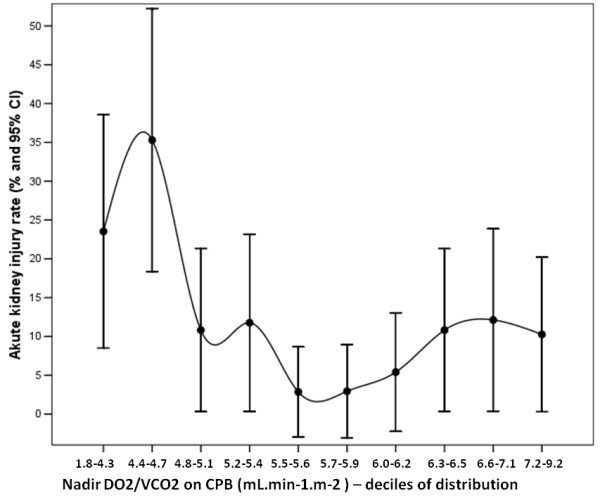
**Acute kidney injury rate according to decile distribution of nadir DO_2_/VCO_2 _ratio during CPB**.

To check the linearity of the relationship between nadir DO_2 _levels and nadir DO_2_/VCO_2 _ratios with postoperative AKI stage 2, we explored the rate of AKI stage 2 outcomes according to the decile distribution of the two independent variables. The graphical analysis of the AKI stage 2 rate according to nadir DO_2 _level and DO_2_/VCO_2 _ratio during CPB (Figures 1 and 2) demonstrated a nonlinearity of the association.

**Figure 3:**
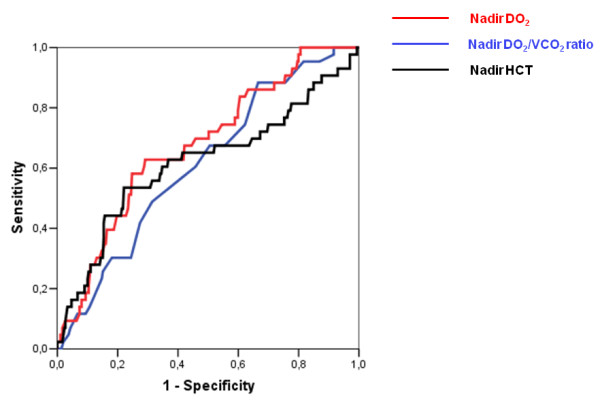
**Receiver operating characteristic curves for acute kidney injury stage 2 rate prediction based on nadir DO_2 _level, nadir DO_2_/VCO_2 _ratio and nadir hematocrit (HCT) level**.

We therefore decided to explore the adequate cutoff values for nadir DO_2 _level and nadir DO_2_/VCO_2 _ratio during CPB as possible predictors of postoperative AKI stage 2 and to include those values in the subsequent multivariable analysis. ROC curves were analysed for nadir DO_2 _levels and DO_2_/VCO_2 _ratios during CPB. To account for the role of the nadir HCT level during CPB (as a component of both of the other variables), this value was included in the analysis (Figure 3).

The best AUC was identified for the nadir DO_2 _level (0.68), with slightly lower values for the nadir DO_2_/VCO_2 _ratio (0.62) and nadir the HCT level (0.61). The best cutoff values according to the Youden's index were set at a nadir DO_2 _< 262 mL/minute/m^2^, a nadir DO_2_/VCO_2 _ratio < 5.3 and a nadir HCT < 23.5%.

The multivariable analysis was performed with AKI stage 2 rate and the rate of any kind of AKI as dependent variables. For each model, nadir DO_2 _value and nadir DO_2_/VCO_2 _ratio were separately analysed because of mathematical coupling between the two variables. The various factors identified in the univariate analysis were tested for intercorrelation. There was a significant intercorrelation between centres and EuroSCORE. Centre 1 included patients at significantly (*P *= 0.001) higher risk for postoperative AKI (EuroSCORE 13.4 ± 17) than centre 2 (EuroSCORE 4.1 ± 2.1). We therefore included the EuroSCORE and not the centre in the multivariable models. Some of the other univariate predictors of AKI are included in the EuroSCORE (age, redo operations and combined coronary and valve operation) and were not included in the model to avoid multicollinearity.

In the multivariable model for AKI stage 2, we tested the following variables: EuroSCORE, CPB duration, number of PRC units transfused, nadir DO_2 _< 262 mL/minute/m^2 ^and nadir DO_2_/VCO_2 _ratio < 5.3. After correction for the other explanatory variables, both a nadir DO_2 _< 262 mL/minute/m^2 ^and a nadir DO_2_/VCO_2 _ratio < 5.3 during CPB remained independently associated with AKI stage 2 rate (Table 3).

**Table 3:** Multivariable stepwise forward logistic regression analysis for acute kidney injury and any damage-independent predictors^**a**^

	Factor		
	
Acute kidney injury severity	β coefficient	Odds ratio (95% CI)	*P *value
Acute kidney injury stage 2			
Model 1			
EuroSCORE	0.034	1.035 (1.016 to 1.054)	0.001
CPB duration (minutes)	0.008	1.008 (1.002 to 1.015)	0.012
Nadir DO_2 _< 262 mL/minute/m^2^	1.135	3.111 (1.531 to 6.319)	0.002
Model 2			
EuroSCORE	0.035	1.036 (1.017 to 1.055)	0.001
CPB duration (minutes)	0.008	1.008 (1.002 to 1.014)	0.007
Nadir DO_2_/VCO_2 _ratio < 5.3	1.062	2.893 (1.452 to 5.763)	0.003
Any acute kidney injury			
EuroSCORE	0.026	1.026 (1.008 to 1.045)	0.005
CPB duration (minutes)	0.009	1.009 (1.003 to 1.015)	0.004
Nadir DO_2 _< 262 mL/minute/m^2^	0.767	2.154 (1.205 to 3.849)	0.010

^a^CPB: cardiopulmonary bypass; CI: confidence interval; DO_2_: oxygen delivery; VCO_2_: carbon dioxide production.

In the multivariable model for any kind of AKI, we tested the following variables: diabetes, EuroSCORE, number of PRC units transfused, CPB duration and nadir DO_2 _during CPB < 262 mL/minute/m^2^. After correction for the other explanatory variables, a nadir DO_2 _< 262 mL/minute/m^2 ^during CPB remained independently associated with the rate of any kind of AKI.

**Figure 4:**
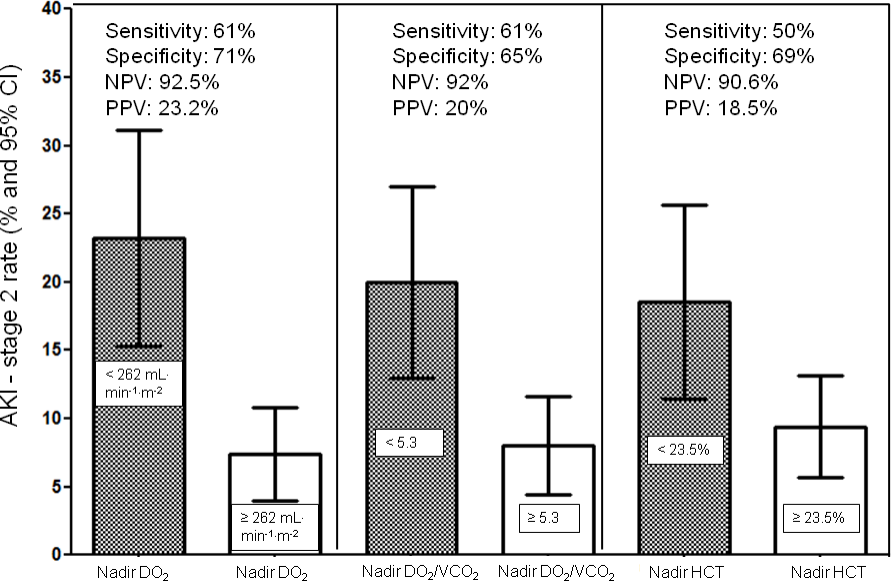
**Acute kidney injury rates in patient groups according to the critical values of DO_2_, DO_2_/VCO_2 _ratio and hematocrit, with sensitivity, specificity, positive predictive value (PPV) and negative predictive value (NPV)**.

The cutoff values identified for the nadir DO_2 _and the nadir DO_2_/VCO_2 _ratio during CPB were investigated for their impact on the clinical patterns of AKI stage 2 (Figure 4). The patient group with a nadir DO_2 _value < 262 mL/minute/m^2 ^during CPB had a significantly (*P *= 0.001) higher rate of postoperative AKI stage 2 (23.2% vs. 7.4%), with a negative predictive value of 92.5%. The patient group with a nadir DO_2_/VCO_2 _ratio < 5.3 during CPB had a significantly (*P *= 0.001) higher rate of AKI stage 2 (20% vs. 8%), with a negative predictive value of 92%. The patient group with a nadir HCT < 23.5% had a significantly (*P *= 0.014) higher rate of AKI stage 2 (18.5% vs. 9.4%).

As a secondary end point, we investigated the association of the three study variables (nadir DO_2_, peak VCO_2 _and nadir DO_2_/VCO_2 _ratio during CPB) with the ICU and postoperative hospital stays using linear regression analysis. We found significant (*P *= 0.019 and *P *= 0.001, respectively) negative associations between ICU length of stay and postoperative hospital length of stay with nadir DO_2 _during CPB. Conversely, there was no association of ICU length of stay and postoperative hospital length of stay with the peak VCO_2 _value and the nadir DO_2_/VCO_2 _ratio (Table 4). The previously identified cutoff value for the nadir DO_2 _maintained its predictive power, being associated with significant differences in both the ICU and postoperative hospital lengths of stay, whereas the cutoff value for the nadir DO_2_/VCO_2 _ratio had only a trend for association with significant differences.

**Table 4:** Univariate association (linear regression and Student's *t*-test) between study variables and ICU and postoperative hospital lengths of stay^**a**^

	Statistical analysis per variable		
Variables	β coefficient	Constant	*P *value
ICU length of stay (days)			
Nadir DO_2 _(mL/minute/m^2^)	- 0.020	8.532	0.014
Peak VCO_2 _(mL/minute/m^2^)	0.034	1.297	0.355
Nadir DO_2_/VCO_2 _ratio	- 0.458	5.703	0.136
Postoperative hospital length of stay (days)			
Factor	β coefficient	Constant	*P *value
Nadir DO_2 _(mL/minute/m^2^)	- 0.055	29.39	0.001
Peak VCO_2 _(mL/minute/m^2^)	0.001	14.15	0.996
Nadir DO_2_/VCO_2 _ratio	- 0.516	17.02	0.428
	Statistical analysis per cutoff value		
	Nadir DO_2 _< 262 mL/minute/m^2^	Nadir DO_2 _≥262 mL/minute/m^2^	*P *value
ICU length of stay (days)	4.2 ± 8.7	2.5 ± 4.4	0.019
Postoperative hospital length of stay (days)	17.6 ± 14.1	12.4 ± 12.1	0.001
	Nadir DO_2_/VCO_2 _ratio < 5.3	Nadir DO_2_/VCO_2 _ratio ≥5.3	*P *value
ICU length of stay length of stay (days)	3.4 ± 6.9	2.9 ± 5.6	0.476
Postoperative hospital length of stay (days)	15.3 ± 13.2	13.4 ± 12.6	0.198

^a^DO_2_: oxygen delivery; VCO_2_: carbon dioxide production.

## Discussion

AKI after cardiac surgery is one of the major determinants of bad outcomes, with increased early and late mortality rates and a considerable consumption of human and financial resources. Many factors are implicated in the complex mechanisms leading to postoperative kidney function impairment, and unfortunately the great majority are due to unmodifiable factors: advanced patient age, preoperative renal dysfunction, diabetes, hypertension, peripheral arteriopathy, chronic lung disease, complex surgery and other factors [2-6,16].

In the present study, we investigated the role of potentially modifiable factors related to CPB surgery in determining postoperative AKI. Our results demonstrate, in a relatively large series of patients treated at different sites, that a low DO_2 _level and/or a low DO_2_/VCO_2 _ratio during CPB are independently associated with postoperative AKI. Given the nature of our study, we cannot confirm a causative relationship between DO_2 _and postoperative renal function. We have identified specific cutoff values for both DO_2 _(262 mL/minute/m^2^) and DO_2_/VCO_2 _ratio (5.3), and for both these values the negative predictive power exceeds 90%. However, the measurement of CO_2_-related variables did not increase the accuracy of the DO_2_-based predictive model. In clinical terms, our data generate the hypothesis that by maintaining the DO_2 _at a level > 262 mL/minute/m^2 ^during CPB, the likelihood of experiencing a postoperative AKI might be decreased.

Moreover, the cutoff value of 262 mL/minute/m^2 ^as the lowest acceptable DO_2 _during CPB is associated with significant differences in terms of ICU and postoperative hospital lengths of stay (about two and five days, respectively).

This study confirms some of the critical values previously identified in other studies [8,12,13]. Values of DO_2 _< 272 mL/minute/m^2 ^during CPB have been associated with an increased rate of acute renal failure [8], and values < 260 mL/minute/m^2 ^have been associated with increased lactate formation [12].

It is well known that below a critical DO_2 _level, O_2 _consumption cannot be maintained using aerobic energy production, and that to provide energy to the cells, the anaerobic mechanism is activated [17,18]. As a consequence, blood lactate concentration starts rising, causing excess proton production, and buffering of the protons by bicarbonate results in increased VCO_2_. This mechanism was identified for kidney tissue in 1966 [19]. Under normothermic conditions in conscious humans [20], the critical DO_2 _level is around 300 mL/minute/m^2^. In our study, we identified and confirmed that under anaesthesia and moderate hypothermia, values as low as 262 mL/minute/m^2 ^can be sustained, but below this value kidney function starts to decline.

The measurement of VCO_2 _-related variables did not increase the accuracy of AKI prediction in our series. VCO_2 _may increase even under normal aerobic conditions, and during CPB this invariably happens during the rewarming phase as a result of increased metabolic needs. Theoretically, if coupled with the DO_2 _measurement (DO_2_/VCO_2 _ratio), the measurement of VCO_2 _should offer additional advantages for detecting dysoxia, as suggested by other authors who demonstrated that CO_2_-related variables, in combination with O_2_-related variables, are good indicators of critical hypoperfusion [21,22]. Under our experimental conditions, no added value of CO_2_-related variables was detected.

The results of our study, linking postoperative AKI to a condition of 'dysoxia' during CPB, strengthen the hypothesis that a condition of inadequate O_2 _supply during CPB might lead to a hypoxic insult to the kidney, as previously hypothesized by other authors who demonstrated the relationship between a low Hb value during CPB and bad renal outcomes [9-11].

Hb content and HCT level are physiological variables that concur in the definition of the DO_2_. As such, they have already been identified as independent predictors of postoperative AKI [9-11]. In our study, we have confirmed that the haemodilutional effects (nadir HCT during CPB) are independently associated with postoperative AKI. The nadir DO_2 _had a marginally higher accuracy in predicting postoperative AKI, as suggested in a previous study [8], but the respective roles of HCT and pump flow in determining postoperative AKI remain to be clarified, and specific randomized trials are required.

Kidney physiology and function are particularly dependent on O_2 _supply. Under normal physiologic conditions, peritubular capillaries are nourished by efferent glomerular arteries [23], which carry poorly oxygenated blood, leading to renal parenchymal hypoxia [24]. This effect is particularly pronounced at the level of the renal medulla, with partial pressure of O_2 _(pO_2_) levels as low as 25 mmHg [25]. Therefore, it is not surprising that the kidney might be one of the first organs to be affected with a global reduction in DO_2_. *In vitro *studies have demonstrated that some highly susceptible areas of the kidney are prone to ischemic injury in cases of even slight reductions in renal DO_2 _[26].

Reduced O_2 _content in cases of acute anaemia is usually compensated by reduced blood viscosity with increased blood flow in the microcirculation and by a compensatory increase in cardiac output. This last mechanism may be impaired during CPB, where pump flow is usually adjusted on the basis of the patient's body surface area and temperature, not the Hb value.

Our study highlights some concepts related to postoperative AKI prevention in cardiac surgery: (1) CPB is one of the major determinants of AKI, and, in our analysis, both CPB duration and the metabolic variables measured during CPB were independent predictors of AKI; (2) opposite to other determinants of postoperative AKI, DO_2 _belongs to the field of the modifiable risk factors; and (3) the negative predictive value of the identified cutoff value is very high, suggesting the potential clinical usefulness of these values within a preemptive strategy of renal protection.

Goal-directed strategies are key to improving artificial heart and lung support during CPB. This is especially true, given our more complex population with more comorbidities than those in other studies. An effective goal-directed strategy should be based on proven, clinically significant quality indicators such as DO_2_. Historically, it has been difficult to deploy CPB goal-directed strategies because of the limitations of parameters such as cardiac index, mean arterial pressure, venous saturation, arterial blood temperature, minimum Hb, partial pressure of CO_2 _and pO_2_.

The take-away message from our results is that the maintenance of adequate DO_2 _may limit the risk of postoperative AKI. To maintain an acceptable DO_2_, the pump flow should be adjusted according to the Hb value of the perfusate, not only according to the patient's body surface area and temperature. In other words, the pump should be used to adjust the cardiac output exactly, as it happens as a compensatory physiological mechanism during acute, severe anaemia.

Our study has limitations. It lacked patients treated under hypothermic conditions < 27°C, when different limits of critical DO_2 _are likely to occur. The study is not powered to detect other organs' responses to low DO_2_. Finally, this study was not a randomized, controlled trial comparing a specific strategy (targeted at a DO_2 _> 262 mL/minute/m^2^) with conventional CPB management.

## Conclusions

The concept of goal-directed CPB perfusion merits further investigations that are based on clinically significant quality indicators such as DO_2_. This concept should be tested for its clinical effects on kidney function and the function of other organs in an adequately large, randomized, controlled trial.

## Key messages

• A nadir DO_2 _< 262 mL/minute/m^2 ^during CPB is independently associated with AKI stage 2 following cardiac operations.

• Other determinants of AKI are patient age, the presence of comorbidities, the type of operation (all included in risk stratification scores such as the EuroSCORE) and CPB duration.

• Nadir DO_2 _level is significantly associated with prolonged ICU and postoperative hospital lengths of stay.

• Goal-directed perfusion aimed at preserving DO_2 _during CPB (by reducing haemodilution and maintaining high pump flow) should be tested as a possible preemptive strategy for postoperative AKI in a randomized, controlled trial.

## Abbreviations

AKI: acute kidney injury; AUC: area under the curve; CPB: cardiopulmonary bypass; DO_2_: oxygen delivery; Hb: haemoglobin; PRC: packed red cells; ROC: receiver operating characteristic; VCO_2_: carbon dioxide production.

## Competing interests

MR is the owner of a patent for DO_2 _and VCO_2 _monitoring during CPB and received honoraria from Medtronic Inc. and Sorin Group for speaking at congresses and symposia. No financial or nonfinancial competing interests exist for any of the other authors.

## Authors' contributions

MR designed the study, performed the statistical analysis and prepared the manuscript. FDS and MB were responsible for data acquisition and interpretation. JM was responsible for data acquisition and interpretation and drafted the manuscript. GVN was responsible for data acquisition and manuscript preparation. TA performed the statistical analysis and designed the study. All authors read and approved the final manuscript.
